# An In Silico Investigation of the Structural and Evolutionary Features of MAPEG Proteins in Bacteria

**DOI:** 10.3390/cimb48090939

**Published:** 2026-09-15

**Authors:** Luca Federici, Michele Masulli, Vincenzo De Laurenzi, Nerino Allocati

**Affiliations:** 1 Department of Innovative Technologies in Medicine and Dentistry, University “G. d’ Annunzio”, I-66100 Chieti, Italy; luca.federici@unich.it (L.F.); vincenzo.delaurenzi@unich.it (V.D.L.); 2 CAST (Center for Advanced Studies and Technology), University “G. d’ Annunzio”, I-66100 Chieti, Italy; 3 Department of Medical, Oral and Biotechnological Sciences, University “G. d’ Annunzio”, I-66100 Chieti, Italy; michele.masulli@unich.it

**Keywords:** bacterial MAPEG proteins, AlphaFold model, molecular docking, CB-Dock2

## Abstract

The Membrane-Associated Proteins in Eicosanoid and Glutathione metabolism (MAPEG) superfamily comprises integral membrane proteins involved in lipid metabolism, detoxification, and redox regulation. While extensively characterised in eukaryotes, bacterial MAPEG homologs remain poorly understood. In this study, we conducted an in silico analysis of bacterial MAPEG proteins and performed a comparative investigation with selected eukaryotic homologs to assess structural conservation and evolutionary relationships. Sequence alignment and phylogenetic reconstruction showed that the bacterial proteins have the typical MAPEG signature motifs, including residues associated with glutathione binding. Homology modelling and structural prediction analyses indicated conservation of the characteristic trimeric transmembrane architecture typical of the MAPEG family. Comparative structural analysis highlighted a preserved core fold across domains of life, accompanied by variations in loop regions and membrane-embedded segments that may reflect adaptation to distinct cellular environments. Molecular docking simulations suggested a conserved glutathione-binding pocket, although subtle differences in the surrounding residues may influence substrate specificity. Overall, our findings support an evolutionarily conserved structural framework within the MAPEG superfamily and provide insight into the diversification of bacterial members relative to their eukaryotic homologs. This study contributes to a deeper understanding of MAPEG protein evolution and lays the groundwork for future functional and biochemical investigations.

## 1. Introduction

**Figure 1:**
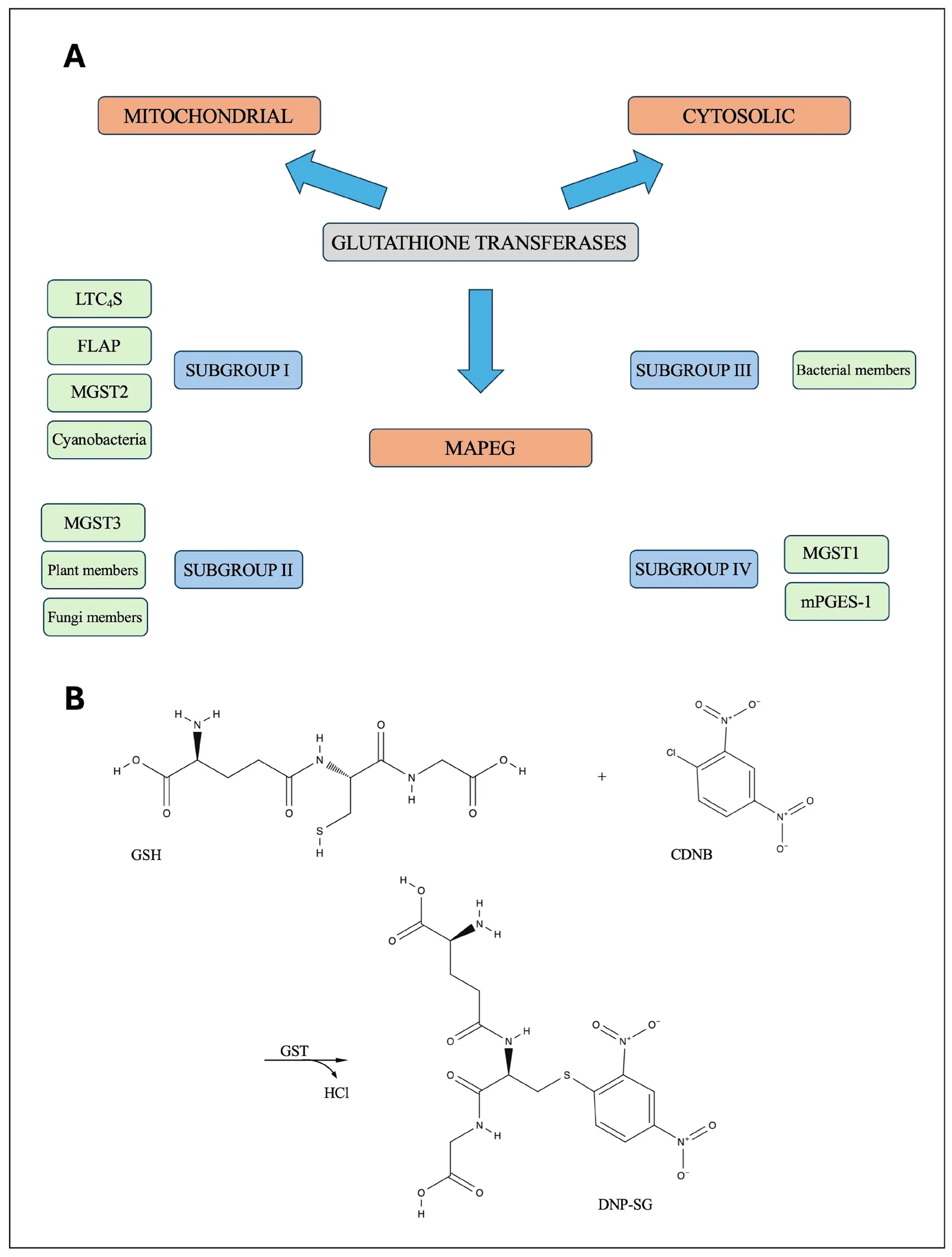
(**A**) Graphical overview of the three GST families. The subdivision of the MAPEG family is based on the amino acid sequence identity and crystallographic structures. (**B**) The conjugation of glutathione (GSH) to 1-chloro-2,4-dinitrobenzene (CDNB) as catalysed by GSTs. This reaction results in the formation of S-(2,4-dinitrophenyl)-glutathione (DNP-SG).

The superfamily of glutathione transferases (GSTs) consists of three distinct families (Figure 1A). The cytosolic and mitochondrial GSTs are soluble enzymes that are distantly related [1,2,3]. MAPEG (Membrane-Associated Proteins in Eicosanoid and Glutathione metabolism), formerly known as microsomal GSTs, is a family of integral membrane proteins involved in several different functions [3,4,5,6]. MAPEG proteins consist of left-handed four-α-helical bundles (TM1–TM4) that assemble into homotrimers [3,7]. Six members have been identified in eukaryotes. Three of them—leukotriene C_4_ synthase (LTC_4_S), microsomal prostaglandin E synthase 1 (mPGES-1) and the arachidonate 5-lipoxygenase activating protein, more commonly referred to as 5-lipoxygenase-activating protein (FLAP)—are involved in the production of arachidonic acid-derivate messengers [8,9,10,11]. Microsomal glutathione transferases (MGST1, MGST2 and MGST3) are primarily implicated in cellular detoxification, mainly through the conjugation of glutathione (GSH) [12,13,14,15,16].

Protein sequence identity, structural properties and enzymatic activities form a basis for the classification of GSTs [3]. MAPEG family has been divided into four subgroups (Figure 1A) [3,4,17]. Among subgroups, MAPEG sequences share less than 20% sequence identity. FLAP, MGST2 and LTC_4_S belong to the first subfamily, while the second subfamily consists of MGST3, and members found in fungi [18] and plants [19]. Members identified in bacteria constitute a distinct third subgroup [4]. Conversely, the GST identified in the cyanobacterium *Synechocystis* (SynMGST) is most closely related to subfamily I [4]. The fourth subfamily is represented by MGST1 and mPGES-1 [1,4,20]. The differences between the four MAPEG subgroups are summarised in Table 1.

Several high-resolution three-dimensional structures are available for cytoplasmic and mitochondrial eukaryotic GSTs [3,21,22] and cytoplasmic prokaryotic GSTs [2,23,24]. As for the MAPEG proteins, solved structures have shown that they are homotrimers, with each monomer containing four trans-membrane alpha helices [3,7,10,25]. A survey of the Protein Data Bank from 17 April 2026 revealed 50 coordinate sets for MAPEG proteins in the complex, with a variety of substrates and inhibitors. The structures were solved for five out of six MAPEG proteins in subgroups I and IV: MGST1 and FLAP, LTC4S and mPGES-1, and MGST2. A GSH-binding site is highly conserved among the classes [3,26] with the exception of FLAP, whose activity is not dependent on GSH [26]. When it is present, all monomers in the trimer have a GSH-binding site, which is located close to the cytoplasmic side of the membrane-spanning regions [3,27]. However, it has been observed that effective GSH binding and subsequent catalytic activity occur at only site at time [13,28,29].

To date, no experimental structure of a bacterial MAPEG is available. Although bacterial MAPEG homologues have been identified and a limited number of proteins have been experimentally characterised, our understanding of their distribution, evolutionary relationships, and molecular properties remains fragmentary. The available studies have mainly focused on individual isolates, such as *Escherichia coli* and *Synechocystis* PCC 6803, and therefore do not provide a broader view of the occurrence and diversification of MAPEG proteins across the prokaryotic domain [5]. In particular, it is unclear whether bacterial MAPEGs are restricted to a few lineages or if they are a more widespread and evolutionarily diverse group. It is also unclear how their sequences and predicted structures relate to the established eukaryotic MAPEG subfamilies. Furthermore, despite the identification of GSH-dependent activity in the previously characterised bacterial proteins, the molecular determinants underlying substrate recognition and ligand binding in bacterial MAPEGs remain poorly defined. The canonical enzymatic activity of GSTs consists of the conjugation of GSH to a wide range of hydrophobic and electrophilic compounds with 1-chloro-2,4-dinitrobenzene (CDNB) commonly used as a model substrate for measuring the enzyme activity of GSTs. All three GST families contain members that catalyse the conjugation of GSH with CDNB to form S-(2,4-dinitrophenyl)-glutathione (DNP-SG) [1] (Figure 1B). Recently, the first in vivo analysis of a cyanobacterial MAPEG (SynMAPEG2, Sll1147, UniProt n° P73795) in *Synechocystis* PCC 6803 was also reported [30]. Cyanobacteria are photosynthetic prokaryotes capable of living in a wide variety of environments [31] and are classified as Gram-negative bacteria, although they have distinctive cell wall features [32]. It has been shown that SynMAPEG2 is dispensable to cell growth in standard photo-autotrophic conditions, while it plays an important role in the resistance to heat and cold. In addition, SynMAPEG2 is involved in the tolerance to n-tertbutyl hydroperoxide, which induces lipid peroxidation, suggesting that SynMAPEG2 may be involved in membrane fluidity, which is critical for photosynthesis [30]. To maintain a consistent nomenclature with the previous literature, we also use the acronym MGST for the bacterial MAPEGs in this paper. Furthermore, the presence of MAPEG proteins in the Archaea domain has not been reported so far [5].

We aimed to identify and investigate previously unrecognised MAPEG proteins in bacteria and Archaea, paying particular attention to their distribution and evolutionary relationships with well-characterised MAPEGs. Specifically, we sought to determine whether bacterial MAPEGs represent a more widespread and diverse group than currently recognised and to assess their relationship with the established MAPEG subfamilies. To this end, we performed an in silico analysis of MAPEG sequences identified across prokaryotic genomes, including both Gram-positive and Gram-negative bacteria as well as Archaea. We analysed the newly identified proteins in terms of their sequence features, predicted structural properties, and phylogenetic relationships. Furthermore, we used molecular docking to investigate the interaction of *E. coli* MGST with GSH and canonical GST substrates. This provided insights into the potential molecular basis of ligand recognition and catalytic activity in bacterial MAPEGs.

## 2. Methods

### 2.1. Transmembrane Topology Prediction

To identify transmembrane (TM) protein topology of four representative bacteria, the TOPCONS web server (https://topcons.net, accessed date on 20 September 2024) with default parameter values was used [33]. TOPCONS is a web server for the consensus prediction of membrane protein topology.

### 2.2. Sequences Analysis and Phylogenetic Relationships

For multiple sequence alignments and phylogenetic analysis, representative MAPEG proteins from each subgroup were obtained from UniProtKB (https://www.uniprot.org/). Non-redundant sequences were selected for bacteria from both Gram-negative and Gram-positive species. In view of the presence of a GST-like protein in Archaea domain [24,34], a new attempt to identify MAPEG proteins in Archaea was undertaken. Three sequences were selected for this domain. The size of the encoded proteins ranges from 119 to 161 residues. This analysis involved 36 amino acid sequences. A multiple-sequence alignment was produced using Clustal Omega software version 1.1.2 (https://www.ebi.ac.uk/jdispatcher/msa/clustalo, accessed on 20 September 2024). The phylogenetic tree was inferred as an unrooted tree using the neighbour-joining statistical method with MEGAX software (version 10.2.6) [35]. The tree is drawn to scale, with branch lengths in the same units as those of the evolutionary distances used to infer the phylogenetic tree. The evolutionary distances were computed using the Poisson correction method and are in the units of the number of amino acid substitutions per site. This analysis involved 36 amino acid sequences. All ambiguous positions were removed for each sequence pair. There was a total of 233 positions in the final dataset. The robustness of the branches was assessed by the bootstrap method with 1000 replications.

### 2.3. Computational Prediction of Three-Dimensional Protein Structure

The three-dimensional structures of bacterial MGST proteins were predicted from their amino acid sequences using AlphaFold3 via the ALPHAFOLD web server (database update on 30 October 2024). AlphaFold3 can predict monomeric and multimeric protein structures with a high degree of accuracy [36]. The quality of the models was evaluated based on the intrinsic confidence estimates provided by AlphaFold3. The following confidence metrics were considered: predicted template modelling (pTM), interface pTM (ipTM), and predicted local distance difference test (pLDDT). A pTM score above 0.5 indicates relatively high confidence in the predicted overall fold of the complex. However, it should be noted that pTM scores are model-derived estimates of structural confidence and do not constitute experimental evidence that the predicted fold corresponds to the native structure. ipTM measures the accuracy of the predicted relative positions of the subunits within the complex. Values above 0.8 represent confident, high-quality predictions. pLDDT is a per-residue measure of local confidence. It is scaled from 0 to 100, with a higher score indicating greater confidence and, consequently, a more accurate prediction [36]. PyMOL software (The PyMOL Molecular Graphics System, Version 3.0 Schrödinger, LLC) was used to generate figures of 3D structures.

The root-mean-square deviation (RMSD) was used to assess the degree of similarity between *E. coli* MGST and two bacterial and two eukaryotic MGSTs. RMSD is a frequently used measure for comparing protein conformations [37]. The calculation of RMSD involves the alignment and optimal superposition of matched pairs of atoms, with the objective of identifying the lowest RMSD result for both structures. The predicted structures were superposed and the RMSDs were calculated using PyMOL. For the comparative analysis, the *E. coli* MGST structure was aligned with the others using the online TM-align tool (version 20190822) [38]. A TM score was computed for each alignment. TM scores can range from 0 to 1, with values close to 1 indicating greater confidence. RMSD and TM scores were then used together to determine structural similarity.

The electrostatic surface potential of *E. coli* MGST was evaluated using the APBS Electrostatics plugin integrated within PyMOL. The protein structure was prepared using the embedded PDB2PQR software to assign atomic charges and radii at pH 7.0 using the AMBER99 force field, while the ligand was kept purely for structural context and visualisation within the binding pocket [39]. The electrostatic potential grid was mapped onto the protein molecular surface, with values visualised from −5 kT/e to +5 kT/e, where red and blue colours represent negative and positive potentials, respectively.

### 2.4. Molecular Docking

Molecular docking was performed to analyse the intermolecular interaction between the *E. coli* MGST protein with GSH and DNP-SG conjugates. Docking simulations were conducted using the CB-DOCK2 online platform [40], which automatically detects potential ligand-binding cavities on the protein surface and performs docking using AutoDock Vina version 1.2.0.

The three-dimensional structure of the homotrimeric *E. coli* MGST model generated by AlphaFold3 was converted into a PDB file using PyMOL software. The protein structure was visually inspected before docking to verify chain integrity and the absence of structural artefacts. The structures of GSH and DNP-SG were retrieved from the PubChem database in SDF format (https://pubchem.ncbi.nlm.nih.gov, accessed on 20 September 2024), which provides pre-optimised 3D chemical geometries, and uploaded directly to the CB-DOCK2 server without further modification. The server automatically handled standard ligand preparation, including geometry refinement and protonation assignment at physiological pH. The docking experiments were repeated three times, and the lowest binding affinity (kcal/mol)—indicating the strongest binding interaction—was selected for each ligand used. The resulting protein–ligand complexes were downloaded and examined with the PyMOL software. Hydrogen bonds (H-bonds) were identified using a distance cut-off of 3.5 Å (mode = 2). All docking calculations were performed using the default parameters of the CB-DOCK2 server. The monomer–monomer interfaces of the predicted *E. coli* MGST trimer were analysed using PyMOL software. The three monomers were examined pairwise (A–B, B–C, and C–A), and interface residues were identified based on intermolecular contacts within a 3.5 Å distance cutoff.

## 3. Results

### 3.1. Transmembrane Topology Prediction

**Figure 2:**
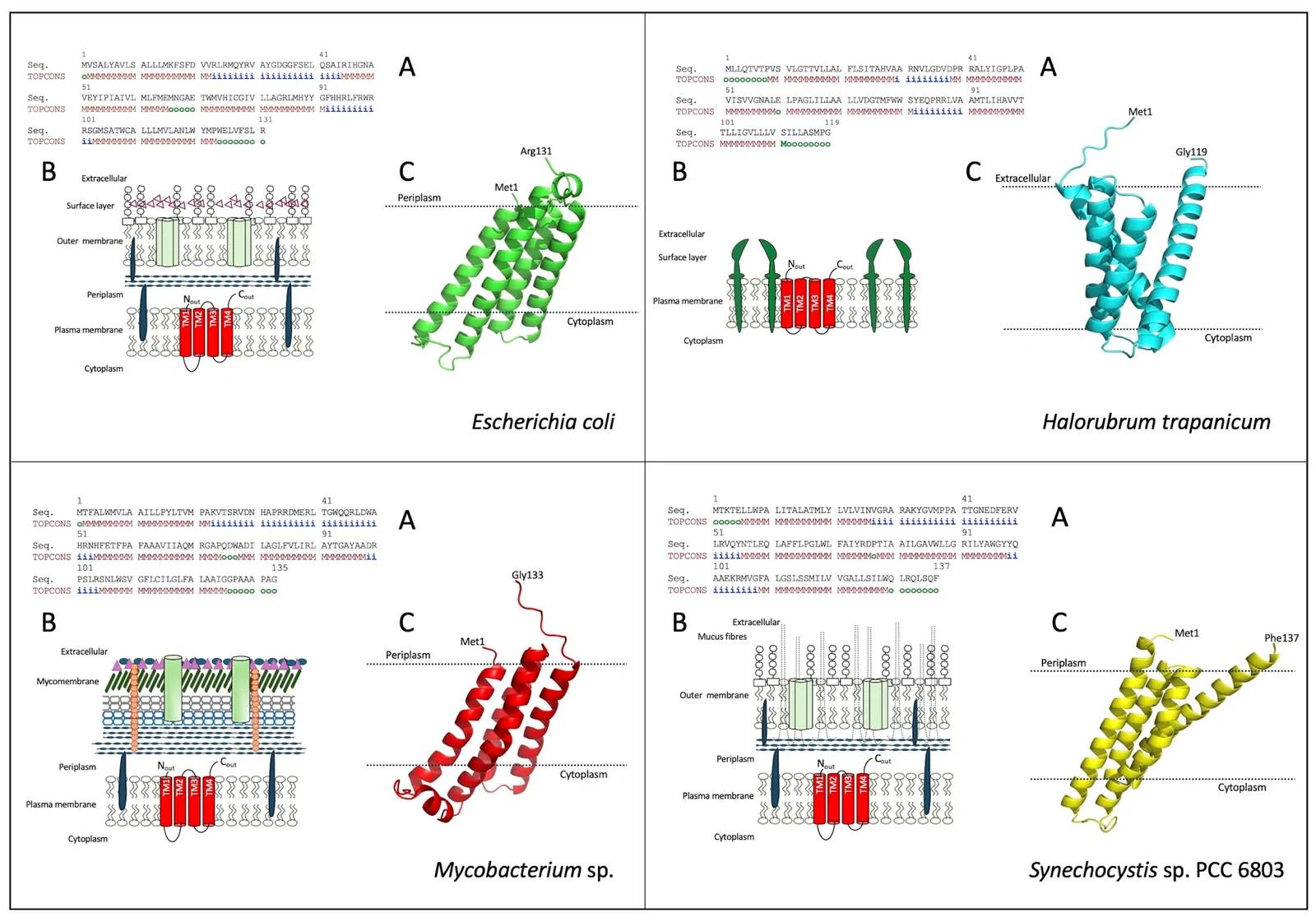
The predicted membrane topology (**A**), the schematic representation of the cell envelope showing the four transmembrane segments (**B**) and the predicted structures of bacterial MGSTs in their monomeric forms from *E. coli* (P64515), *H. trapanicum* (A0A8J7RC23), *Mycobacterium* sp. (UPI00110FEEE7), and *Synechocystis* sp. PCC 6803 (P73795) (**C**). As for the TOPCONS predicted membrane topology: i: stands for inside the membrane; o: outside of the membrane, (M: membrane region). In panels C, the approximate location of the membrane around the membrane-spanning part of the protein is shown by dotted horizontal bars.

Four representative MAPEG protein sequences from different groups of bacteria were used: Gram-negative *E. coli*, Gram-positive *Mycobacterium* sp., the archaea *Halorubrum trapanicum*, and the cyanobacterium *Synechocystis* sp. PCC 6803. All proteins showed the characteristic TM structure of eukaryotic MAPEGs. The monomer comprises four TM helices, which are analogous to those observed in eukaryotes (Figure 2A) [3]. Additionally, as with some eukaryotic proteins, the N- and C-terminal ends are predicted to be located on the external surface of the plasma membrane (Figure 2B,C) [7,9].

The membrane topology predicted by TOPCONS was consistent with the corresponding AlphaFold structures for all four proteins examined. The AlphaFold models confirmed the presence of four transmembrane α-helices and the same-side orientation of the N- and C-termini, thus supporting their predicted localization on the external surface of the plasma membrane (Figure 2C).

### 3.2. Sequences Analysis and Phylogenetic Relationships

**Figure 3:**
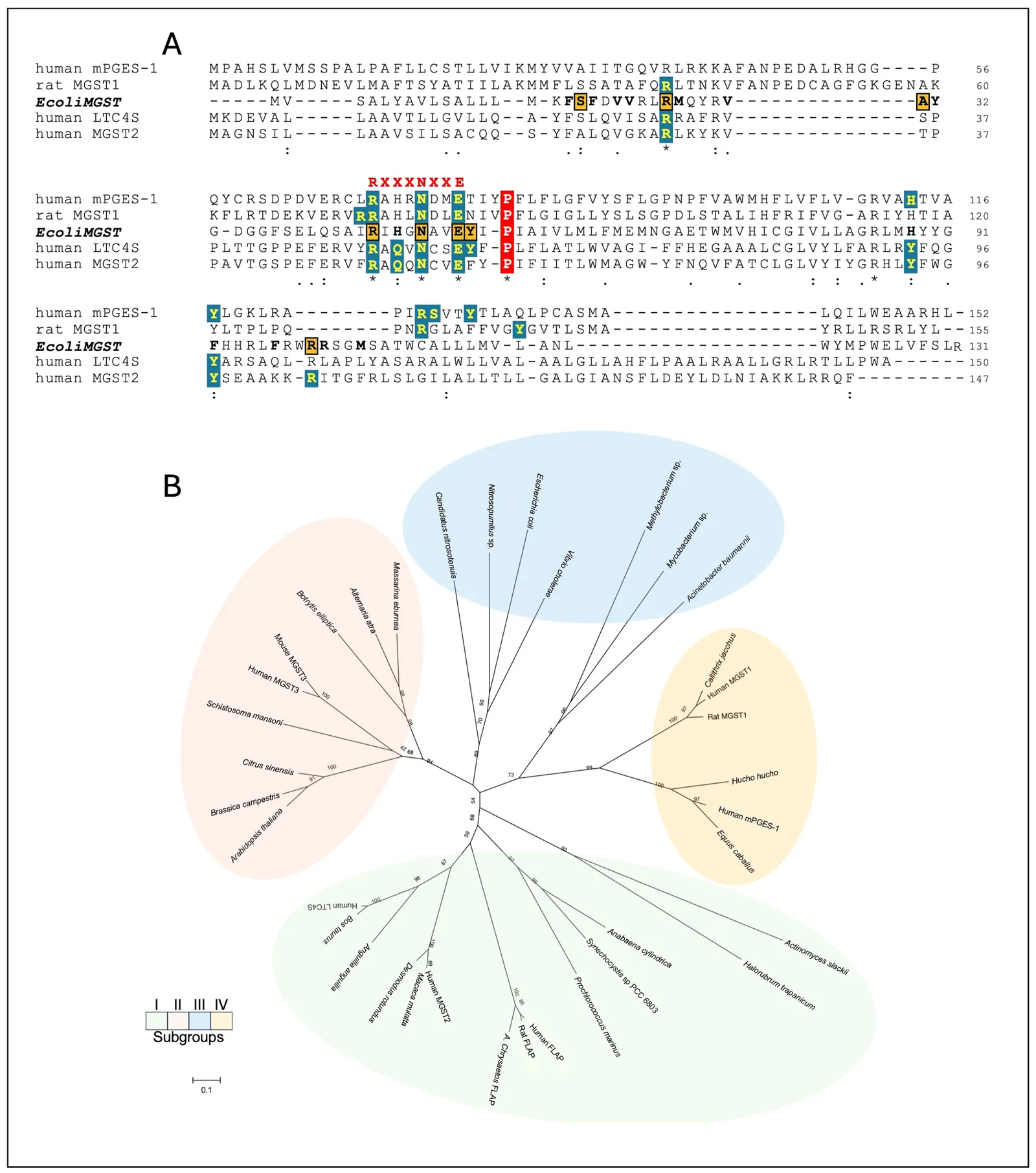
(**A**) Multiple sequence alignment of 36 representative members of the eukaryotic MAPEG family compared with *E. coli* MGST. The motif RX_3_NX_2_E/D is shown in red and bold. Proline residues are marked in red boxes. The conserved residues at the G-site (GSH binding) of eukaryotic MAPEGs are highlighted in teal boxes. The conserved residues at the G-site of the *E. coli* MGST are shown in orange boxes. In black bold are shown the other residues of the *E. coli* MGST binding site. Human mPGES-1 (PDB: AL0), rat MGST1 (PDB: 5I9K), *E. coli* MGST (P64515), human LTC4S (PDB: 2UUH), human MGST2 (PDB: 6SSR). Asterisk (*), colon (:) and period (.) indicate identical, conserved substitution and semi-conserved substitution, respectively. (**B**) Phylogenetic evolutionary analysis of representative members, both prokaryotic and eukaryotic, of the four different MAPEG subgroups. The sequences share the following accession numbers: **subgroup I**: *Anabaena cylindrica* (K9ZND6), *Prochlorococcus marinus* (A2C8J4), *Synechocystis* sp. PCC 6803 (P73795), *Halorubrum trapanicum* (A0A8J7RC23), *Actinomyces slackii* (A0A3S4SKI1), *Bos taurus* (LTC4S, Q2NKS0), human (LTC4S, Q16873; PDB: 2UUH), *Anguilla anguilla* (LTC4S, A0A9D3MQK1), human (FLAP, P20292), rat (FLAP, P20291), *Aquila chrysaetos* (FLAP, A0A663FDG9), human (MGST2, Q99735; PDB: 6SSR), *Desmodus rotundus* (MGST2, K9IGY2) *Macaca mulatta* (MGST2, H9Z3U0); **subgroup II**: *Alternaria atra* (A0A8J2I5C2_9PLEO), *Massarina eburnea* (A0A6A6RU66_9PLEO), *Botrytis elliptica* (MGST3, A0A4Z1J912_9HELO), *Citrus sinensis* (MGST3, A0A067F272_CITSI), *Arabidopsis thaliana* (Q9SHX5), *Brassica campestris* (MGST3, A0A398A9N0_BRACM), human (MGST3, O14880), mouse (MGST3, Q9CPU4), *Schistosoma mansoni* (MGST3, A0A5K4FCX1); **subgroup III**: *E. coli* (P64515), *V. cholerae* (Q9KNE3), *Methylobacterium* sp. (A0A0S9QDY8), *Mycobacterium* sp. (UPI00110FEEE7), *Acinetobacter baumannii* (D0CB87_ACIB2), *Nitrosopumilus* sp. (MDH5666057), *Candidatus nitrosotenuis* (HXE73230); **Subgroup IV**: human (mPGES-1, O14684; PDB: AL0), *Equus caballus* (mPGES-1, Q8HZJ2), *Hucho hucho* (mPGES-1, A0A4W5LIY6), *Rattus norvegicus* (MGST1, P08011; PDB; 5I9K), *Callithrix jacchus* (MGST1, U3FR99), human (MGST1, P10620). Colour coding of subgroups: I, green; II, pink; III, blue; IV, orange.

It has been previously demonstrated that the motif RX_3_NX_2_E/D observed at TM helix 2 is strictly conserved in a number of different MAPEG proteins [12]. The motif is present in all members of the four subgroups, including bacteria (Figure S1). Figure S1 shows the multiple sequence alignments of the full-length sequences analysed in this study. In *E. coli* MGST, the motif is composed by residues Arg45, Asn49 and Glu52 in TM helix 2 (Figure 3A and Figure S1). Some bacterial sequences, as well as the eukaryotic FLAP sequences, lack the arginine residue that is located further away from the motif. Structural studies indicated that the three conserved amino acids of this motif are involved in the interaction with GSH [9,12,41].

In the second TM helix of human mPGES-1 and human MGST2, a proline residue gives rise to a slight kink located in the luminal half of the helix [7,10]. This amino acid residue is highly conserved throughout the MAPEG family (Figure S1). In *E. coli* MGST, it is located at position 55 in the TM helix 2. Proline is typically considered as a helix breaker in soluble globular proteins and it is frequently observed in TM domains [42,43]. It has been observed that the proline residue associated with hydrophobic residues forms a stable motif at the N-terminus of TM helices. Moreover, the high frequency of proline residues in the central segments of TM helices suggests that they may play a structural and/or functional role in forming a hinge in the middle of the TM helix [43,44].

Structural studies of different classes of MAPEG proteins have revealed that many of the amino acids involved in the GSH-binding site are highly conserved [7,9,10,25,41]. A comparison of the sequences of crystallographically solved MAPEG proteins and *E. coli* MGST shows that these conserved residues are also present in the bacterial protein (Figure 3A). Docking analysis confirms these findings (see the molecular docking analysis paragraph).

A phylogenetic analysis using representative prokaryotic and eukaryotic members of the four subgroups is shown in Figure 3B. The phylogenetic relationship among subgroups is consistent with previous classifications, which indicated that bacterial MAPEGs are distributed in subgroups I and III (Figure 1A). The SynMGST branches with the cluster of LTC4S, FLAP and MGST2, while *E. coli* MGST is closely related with other bacteria. An intriguing result of the phylogenetic analysis is the atypical placement of the *A. slackii* and *H. trapanicum* MAPEG-like sequences, which clustered with cyanobacterial homologs rather than with bacterial subgroup III. In particular, the occurrence of a MAPEG family member in *H. trapanicum* is noteworthy, as proteins of this family have not previously been reported in the Archaea domain (Figure 3B).

### 3.3. Structural Analysis

Despite the considerable sequence differences between the various MAPEG proteins, the three-dimensional structures of the monomers were all found to exhibit notable similarities. Accurate predicted structures of bacterial MAPEGs were generated using the AlphaFold3 algorithm [36] (Figure 2C).

The pTM scores obtained for *E. coli* MGST (0.90), *Synechocystis* MGST (0.90), *Mycobacterium* MGST (0.86), and *H. trapanicum* MGST (0.82) indicate a high confidence in the predicted overall folds, as determined by the AlphaFold3 prediction method. However, these scores should not be considered direct evidence that the predicted structures accurately reproduce the corresponding native structures, particularly in the absence of experimental structural or biochemical validation. The pLDDT values ranged from 70 to 90, indicating a high degree of confidence in the predicted local structural features according to the AlphaFold3 model. Nevertheless, both the pTM and pLDDT scores should be interpreted as measures of prediction confidence rather than as direct evidence of structural accuracy.

**Figure 4:**
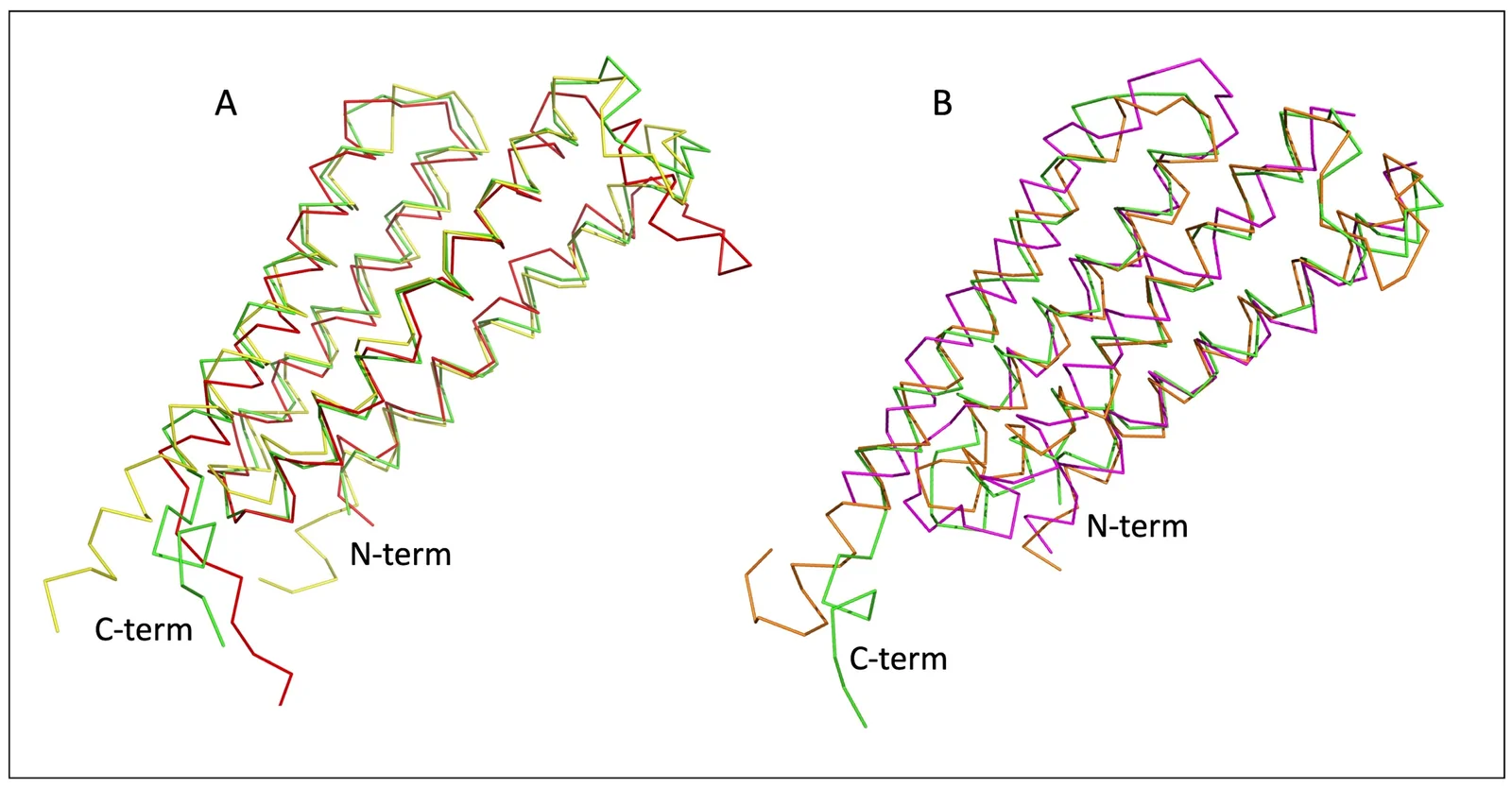
Structural superpositions of MAPEGs. (**A**): *E. coli* MGST (green), *Mycobacterium* sp. (red), and *Synechocystis* sp. PCC6803 (yellow); (**B**): *E. coli* MGST (green), chain B of human MGST2 (orange) and rat MGST1 (magenta). PDB codes: human MGST2 (PDB 6SSR); rat MGST1 (PDB 5I9k) [7,25].

The *E. coli* MGST structure was compared with two others bacterial MAPEGs (Figure 4A). The structures exhibited superposition with the following RMSD values: *E. coli* MGST vs. *Mycobcterium* = 2.659 Å, *E. coli* MGST vs. SynMGST = 6.524 Å. The *E. coli* MGST structure was also superposed with rat MGST1 (PDB: 5I9k) and human MGST2 (PDB: 6SSR) with RMSD values of 3.843 and 1.663 respectively (Figure 4B). It is important to note that the superposition of SynMGST with human MGST2 demonstrated a good result, with an RMSD value of 0.760 Å. This finding is consistent with the hypothesis that SynMGST belongs to subgroup I. In addition, structural comparisons using the TM-align programme revealed a high degree of similarity between *E. coli* MGST and all analysed structures, with TM-scores ranging from 0.68543 to 0.83237. The greatest similarity was observed with SynMGST (TM-score = 0.83237) and human MGST2 (TM-score = 0.82765), followed by *Mycobacterium* (TM-score = 0.77881) and rat MGST1 (TM-score = 0.68543). In agreement with the RMSD results, the TM-align analysis confirmed a particularly high structural similarity between SynMGST and human MGST2, with a TM-score of 0.9072.

**Figure 5:**
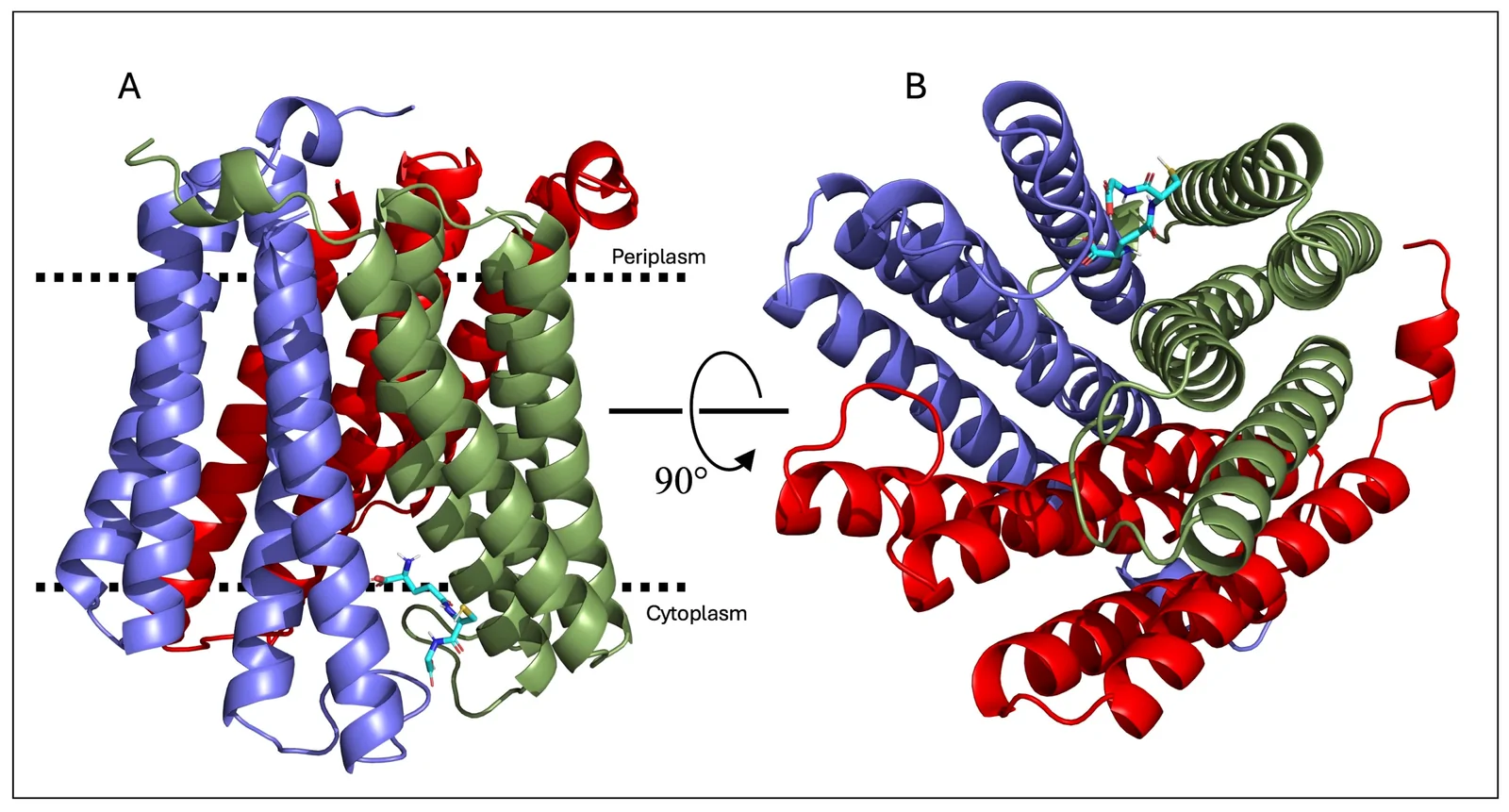
AlphaFold3-derived structure of *E. coli* MGST in its trimeric form. (**A**) The front view with the approximate location of the membrane shown by dotted horizontal bars. (**B**) The view from the cytoplasm. GSH is shown in sticks, as predicted by molecular docking analysis. Monomers of the trimer are coloured in smudge (chain A), red (chain B), and slate (chain C).

Since MAPEG proteins are characterised by a homotrimeric structural configuration, AlphaFold3 was also used to test whether the prokaryotic *E. coli* MGST could assemble as a trimer. An accurate predicted structure was generated, with pTM and ipTM values of 0.84 and 0.86, respectively. The pLDDT values were found to be in the range of 90 > pLDDT > 70. The homotrimer obtained is consistent with crystallographic results obtained for eukaryotic MAPEGs (Figure 5) [3,7,10,12,25].

### 3.4. Molecular Docking Analysis

The available structural data lend support to the hypothesis that the GSH-binding site is highly conserved among MAPEG proteins, and that they utilise a similar mechanism for its activation [7,10,12,25].

**Figure 6:**
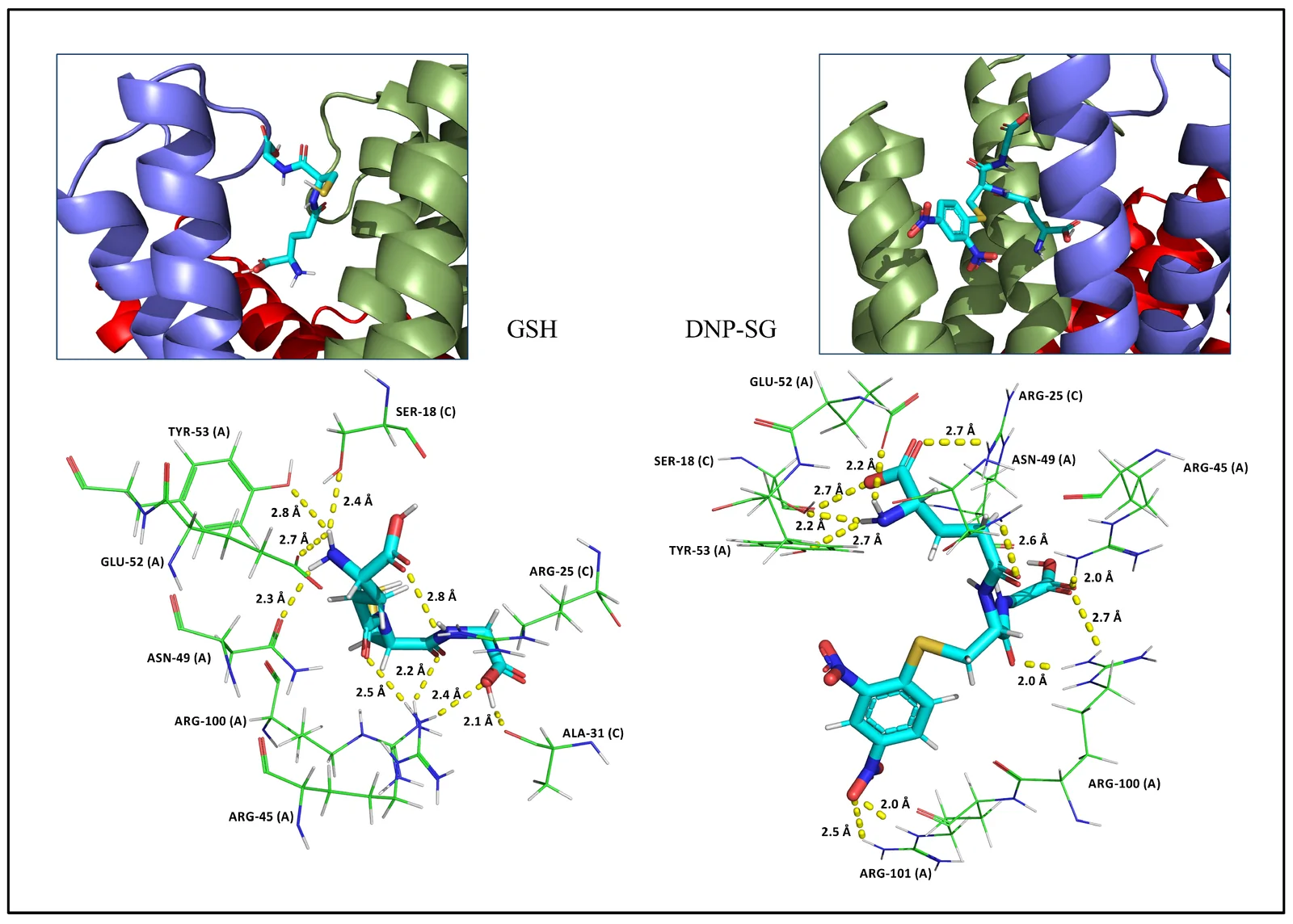
Prediction of GSH (**left**) and DNP-SG (**right**) binding to *E. coli* MGST using molecular docking. GSH and DNP-SG are shown as sticks and the relevant hydrogen bonds with *E. coli* MGST residues are depicted as yellow dashed lines. The corresponding distances are also shown. The monomer involved in the binding is indicated in brackets. Monomers are coloured in smudge (chain A), and slate (chain C).

It has been demonstrated that *E. coli* MGST exhibits GST activity with GSH and CDNB as substrates in the membrane fraction derived from bacterial cells overexpressing the protein [5]. GSTs catalyse the conjugation of GSH with CDNB to form DNP-SG (Figure 1B). Consequently, a molecular docking analysis was conducted to assess the potential binding of both GSH or DNP-SG to *E. coli* MGST. Table 2 shows the data obtained from CB-DOCK2. The putative binding affinity of the GSH and DNP-SG to the protein are −6.0 and −7.8 kcal/mol, respectively. The putative GSH binding site of *E. coli* MGST consists of 21 amino acids: 10 of the chain A and 11 of the chain C (Table 2). GSH in engaged through nine H-bonds involving the side chains of Arg45, Asn49, Glu52, Tyr53 and Arg100 from the chain A, and the side chains of Ser18, Arg25 and Ala31 from the chain C (Figure 6). Additionally, four hydrophobic and five ionic interactions are present.

In the presence of DNP-SG, the binding site consists mainly of the same amino acid residues as GSH. In chain A, there are two additional amino acids involved (Ser105 and Trp108), while in chain C, Tyr32 is missing (Table 2). Furthermore, the substrate is engaged through eleven hydrogen interactions with eight residues of the chain A and three of the chain C (Figure 6). The presence of six hydrophobic interactions and five ionic integrations is also observed.

**Figure 7:**
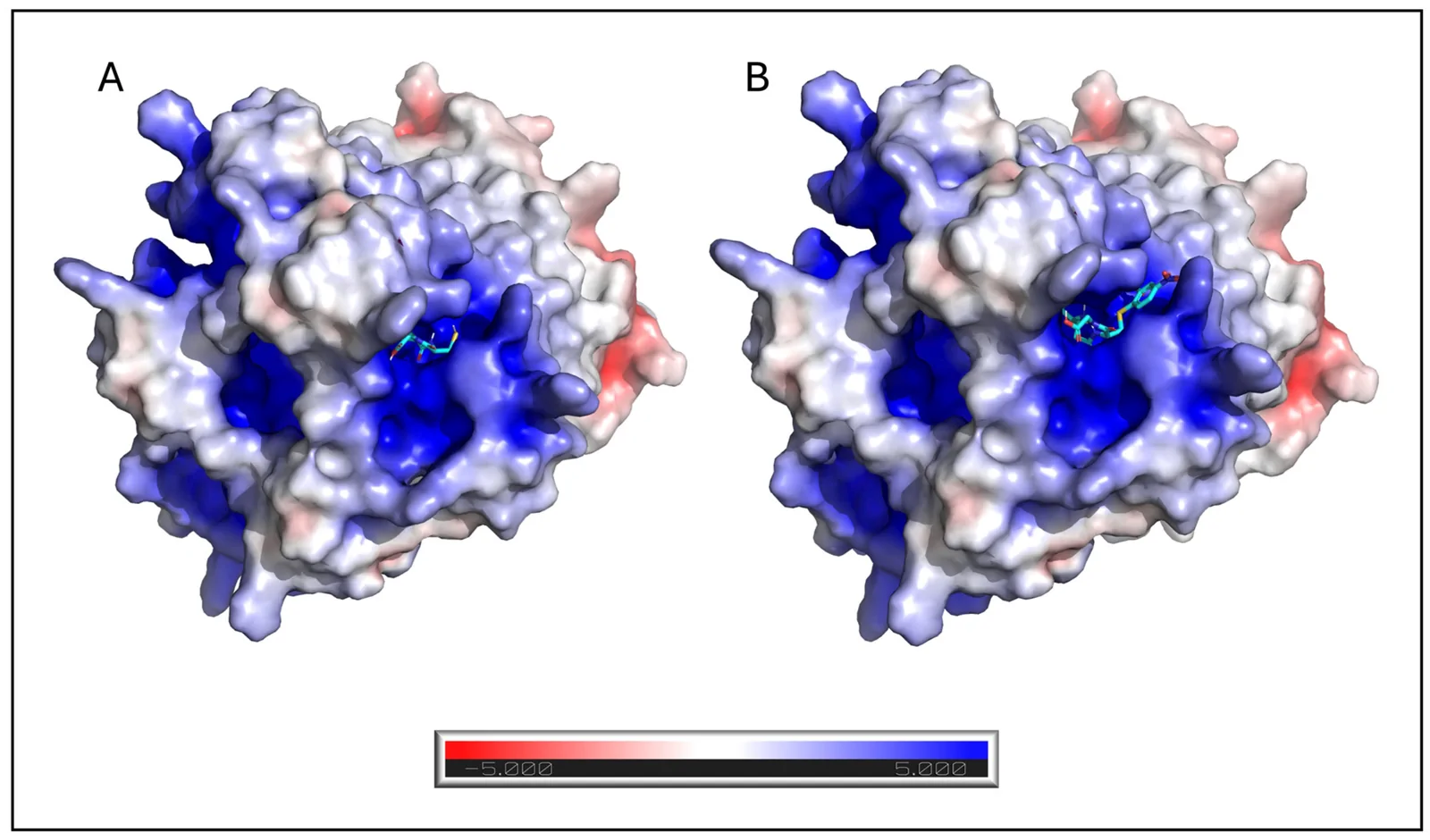
APBS-calculated electrostatic potential surfaces of *E. coli* MGST with GSH (**A**) and DNP-SG (**B**) bound in the active site. Red indicates regions of negative electrostatic potential, whereas blue indicates regions of positive electrostatic potential.

It has been shown in eukaryotic MAPEGs that the three conserved amino acids of the RX3NX2E/D motif play a role in ligand binding [12]. In *E. coli* MGST, molecular docking analysis suggested that the three conserved amino acids of this motif may interact with both GSH and DNP-SG. Arg45, Asn49 and Glu52 interact with GSH with H-bond lengths of 2.5 and 2.4, 2.3 and 2.7 Å, respectively (Figure 6). Arg45, Asn49 and Glu52 also interact with DNP-SG with H-bond lengths of 2.0, 2.6 and 2.2 Å, respectively (Figure 6). Although standard parameters were used (three runs), the proposed binding mode was further supported by structural alignment with eukaryotic homologues (Figure 4). This geometric convergence lends weight to the idea of pocket conservation and the biological plausibility of the top-ranked poses. To investigate the structural determinants that contribute to trimer formation, the monomer–monomer interfaces of the *E. coli* MGST model were analysed. Due to the high structural symmetry of the homotrimer, the amino acid residues involved in stabilising the interfaces are identical across all three interacting pairs (A–B, B–C, and C–A). Specifically, the interface between adjacent subunits is stabilised by a network of reciprocal interactions involving Ala31, Tyr53, Glu70, and Asn67 from one monomer, and Arg45, Ser18, Arg131, and Met1 from the neighbouring subunit. Notably, some of these key interface-stabilising residues overlap directly with the amino acids involved in the dynamic binding and accommodation of GSH (Table 2). This robust and identical residue conservation across all three structural interfaces strongly supports the existence of a conserved, evolutionarily shared mechanism for trimeric assembly, in which structural stabilisation and active site architecture are strictly interdependent. To further characterise the binding pocket, electrostatic potential surface representations were generated for *E. coli* MGST in complex with GSH and DNP-SG (Figure 7). These electrostatic surfaces provide a visual representation of the charge distribution within the binding site, enabling comparison of the electrostatic environments associated with the two ligands.

## 4. Discussion

In this study, we investigated the structural and functional features of bacterial MAPEG proteins, with a focus on *E. coli* MGST, and compared them with representative members from various other prokaryotic groups. Despite sequence variability, the analysed proteins displayed a conserved four-transmembrane helix topology, which is consistent with the architecture reported for eukaryotic MAPEG proteins. In contrast, the predicted orientation of both the N- and C-termini towards the extracellular side of the membrane is only observed in some eukaryotic MAPEG proteins [7]. The GSH-binding site is located at the interface between adjacent monomers, near the cytoplasmic side of the predicted transmembrane regions (Figure 5). The location of the binding site is similar to those observed for other MAPEG proteins [25].

A key finding of the sequence analysis is the strict conservation of the RX_3_NX_2_E/D motif within TM helix 2. In *E. coli* MGST, residues Arg45, Asn49 and Glu52, which belong to this motif, were suggested by molecular docking to participate directly in ligand binding. These residues form hydrogen bond interactions with both GSH and DNP-SG, which supports their role in substrate recognition and stabilisation. This finding is also consistent with previous structural studies and highlights the functional importance of this motif in MAPEG proteins.

The presence of a conserved proline residue within TM helix 2 introduces a local distortion that may contribute to functional dynamics. Proline-induced kinks are often linked to conformational flexibility in membrane proteins and could facilitate the transitions necessary for ligand binding or product release. The conservation of this residue across the MAPEG family suggests that it plays a structural role linked to function. Because MAPEG proteins are integral membrane proteins, the surrounding lipid bilayer may influence substrate accessibility, protein dynamics, and the conformation of catalytically relevant structural elements, including the conserved Pro55 kink. Consistent with this view, Kuang et al. [25] demonstrated that specific lipid–protein interactions contribute to the structural organisation of the eukaryotic MGST1. Although bacterial MAPEG proteins differ in their lipid requirements and regulatory mechanisms, these findings highlight the importance of considering the membrane environment when interpreting computational docking results. Therefore, the ligand-binding modes proposed here should be regarded as plausible structural hypotheses that nevertheless require further validation. The topology of the phylogenetic tree is consistent with the possibility that the evolutionary history of this MAPEG protein family may have involved ancient horizontal gene transfer (HGT) events between bacterial and archaeal lineages. Rather than forming a distinct ancestral lineage, archaeal homologues appear to be polyphyletic. *C. nitrosopumilus* sp. clusters within subgroup III of the bacterial lineage, while *H. trapanioum* is positioned within subgroup I. This positioning could be explained by independent inter-domain HGT events from bacterial lineages potentially co-occurring in shared ecological niches. However, the present phylogenetic evidence alone is insufficient to conclusively establish HGT, and alternative evolutionary scenarios cannot be excluded.

The high structural similarity between predicted bacterial MAPEG models and eukaryotic structures strongly supports the idea of divergent evolution from a common ancestor, rather than convergent evolution. Despite low sequence identity, the absolute conservation of the four-transmembrane helix fold and the highly conserved three-dimensional spatial arrangement of GSH-binding residues rule out convergence. In an evolutionary context, subgroup III forms a well-supported monophyletic clade, diverging cleanly from the two eukaryotic subgroups, as well as the mixed lineages of subgroup I. This divergence suggests a specialised functional evolution within bacterial subgroup III. The presence of key human pathogens and environmental colonisers within subgroup III hints at an evolutionary pressure to adapt this MAPEG protein family for specific metabolic or physiological tasks, such as protection against oxidative stress during host colonisation or environmental transitions.

The structural predictions obtained showed high confidence scores and revealed a conserved fold among bacterial and eukaryotic MAPEG proteins. Structural superposition analyses yielded low RMSD values, indicating a high degree of structural similarity despite limited sequence identity. These findings suggest that the overall fold is highly conserved and is probably the basis of a shared catalytic mechanism. Furthermore, the high TM-scores obtained from the structural comparisons provide additional evidence of the overall structural similarity of *E. coli* MGST to the analysed MGST proteins. This includes the particularly high level of similarity observed with SynMGST and human MGST2.

Consistent with known MAPEG structures, *E. coli* MGST was predicted to assemble as a homotrimer. This oligomeric state is functionally relevant, as the active site of MAPEG proteins is located at the subunit interface. The predicted trimeric organisation therefore supports the formation of a competent catalytic site, which is consistent with crystallographic data from eukaryotic homologues.

Further molecular docking analyses support the functional role of *E. coli* MGST as a GSH-dependent enzyme. The predicted binding affinities for GSH and DNP-SG suggest stable interactions, and the involvement of conserved residues in hydrogen bonding and electrostatic interactions indicates the presence of a well-defined binding site. These results are consistent with previously reported GST activity in bacterial systems, suggesting that *E. coli* MGST may contribute to detoxification processes through GSH conjugation. Overall, these findings suggest that bacterial MAPEG proteins possess the essential structural and functional characteristics of the superfamily, including conserved topology, catalytic residues, and oligomeric organisation.

In conclusion, the conservation of the RX_3_NX_2_E/D motif, overall fold and trimeric assembly suggests a potentially shared catalytic mechanism with eukaryotic homologues. These findings provide further evidence of the evolutionary conservation of MAPEG proteins and reinforce their role in cellular detoxification pathways.

Despite the predictive capabilities of the AlphaFold3 and CB-DOCK2 algorithms, computational predictions of protein–ligand interactions inevitably entail a degree of uncertainty. Similarly, AlphaFold3-predicted structures should be interpreted with caution in the absence of experimental validation, particularly for membrane proteins and multimeric interfaces. Furthermore, it must be acknowledged that the docking simulations performed in this study provide static binding poses. Since MAPEG proteins are membrane-bound complexes characterised by dynamic substrate binding and conformational flexibility during catalysis, the static nature of our computational models is limiting. While these homology-validated poses provide a robust predictive basis, future molecular dynamics simulations will be crucial to fully elucidate the structural dynamics and conformational transitions of *E. coli* MGST within a lipid bilayer environment. To substantiate the proposed interactions, experimental validation will be necessary, including kinetic analyses and site-directed mutagenesis of key residues. Therefore, future studies integrating structural and biochemical approaches are required to elucidate the catalytic mechanism and, more importantly, to identify the physiological substrates of bacterial MAPEG proteins. This will enhance our understanding of their biological function.

## Figures and Tables

**Table 1:** A schematic overview of the four MAPEG proteins subgroups. The classification, enzymatic properties, and biological roles of the four distinct subfamilies within the MAPEG superfamily.

Subgroup	Representative Members	Biochemical Activity	Main Biological Functions	Refs.
Subgroup I	LTC4S	Leukotriene C4 synthase	Leukotriene C4 biosynthesis	[5,9,17]
	FLAP	Binds to 5-LOX ^1^	Activation of 5-lipoxigenase	[5,11,17]
	MGST2	LTC4 synthase, GST, GSH Peroxidase	Leukotriene C4 biosynthesis defence against xenobiotics response to oxidative stress	[5,15,16,17]
	Cyanobacteria	GST, GSH-dependent redox process	Defence against xenobiotics response to oxidative stress	[5]
Subgroup II	MGST3	GST, LTC4 synthase, GSH Peroxidase	Defence against xenobiotics response to oxidative stress leukotriene C4 biosynthesis	[5,14,16,17]
	Plants	GST, binding and transport.	Response to oxidative stress Conjugation and transport into the vacuole	[5,19]
	Fungi	GST, GSH Peroxidase	Defence against xenobiotics response to oxidative stress	[18]
Subgroup III	*E. coli* MGST	GST	Defence against xenobiotics	[5]
Subgroup IV	MGST1	GST, GSH Peroxidase	Defence against xenobiotics Response to oxidative stress	[5,14,17]
	mPGES-1	GSH-dependent oxidoreductase	Oxidoreduction of PGH_2_^2^ into PGE_2_	[5,10,17]

^1^ 5-lipoxygenase (5-LOX) catalyses the first step in the leukotriene synthesis. ^2^ Prostaglandin endoperoxide H.

**Table 2:** Predicted active site residues of *E. coli* MGST in the presence of GSH or DNP-SG. Conserved residues are indicated in bold.

Substrate	Vina Score (kcal/mol)	Cavity Volume (Å^3^)	Docking Size (Å) (x,y,z)	Binding Site Residues
GSH	−6.0	1107	23,23,23	**Chain A**: **Arg-45**, **Asn-49**, **Glu-52**, **Tyr-53**, His-88, Phe-92, Phe-97, **Arg-100**, Arg-101, Met-104 **Chain C**: Phe-17, **Ser-18**, Phe-19, Val-21, Val-22, **Arg-25**, Met-26, Val-30, **Ala-31**, Tyr-32, His-47
DNP-SG	−7.8	1107	25,25,25	**Chain A**: **Arg-45**, **Asn-49**, **Glu-52**, **Tyr-53**, His-88, Phe-92, Phe-97, **Arg-100**, **Arg-101**, Met-104, Ser-105, Trp-108 **Chain C**: Phe-17, **Ser-18**, Phe-19, Val-21, Val-22, **Arg-25**, Met-26, Val-30, Ala-31, His-47

## Data Availability

The original contributions presented in the study are included in the article/Supplementary Materials, further inquiries can be directed to the corresponding author.

## Supplementary Materials

The following supporting information can be downloaded at: https://www.mdpi.com/article/10.3390/cimb48090939/s1.

## Abbreviations

The following abbreviations are used in this manuscript:

MAPEG	Membrane-Associated Proteins in Eicosanoid and Glutathione metabolism
GSH	Glutathione
CDNB	1-chloro-2,4-dinitrobenzene
