# Supplementary material for: An In Silico Investigation of the Structural and Evolutionary Features of MAPEG Proteins in Bacteria
Source: Curr Issues Mol Biol. 2026 Sep 15;48(9):939. doi: 10.3390/cimb48090939 (PMC13605077; doi:10.3390/cimb48090939)
Supplement: Supplementary file 1 [file cimb-48-00939-s001.zip › cimb-4502757-supplementary.pdf]

```
                              1MMMMMMMMMMMMMM       MMMMMM1                          2MMM
Escherichia coli (EcoliMGST) MVSALYAV-LSALLLM-------KFSFDVVRLRMQYRVAYG--DGGFSE----LQSAIRI 46
Methylobacterium sp.         MP---VEIRLLAWSCFLGLVHIIAASYSGLQQRGGFTWAAGNREGRTPEVTGAAARLEKA 57
Vibrio cholerae              MVTTGYAVVLCGWLI--------YLAVQVIRQRRKHQVLFA--DGGVDA----LVRARSA 46
                             *      : *               : . :: *  .    .  :*
                             MMMMMMMMMMMMMMMMMM2     3MMMMMMMMMMMMMMMMMMMMM3           4M
Escherichia coli (EcoliMGST) HGNAVEYIPIAIVLMLFMEMNGAETWMVHICGIVLLAGRLMH--YYGFHHRLFRWRRSGM 104
Methylobacterium sp.         SKNFFETFPIFAALVLACVATGRHNTAVVLGAYLYFFGRLAYLPVYGLGVPKI--R---- 111
Vibrio cholerae              QSNATEYIPIFLILLGLAEMNGVNVWWIHALGVAFVVGRVLH--ADSMFKATIPNRVRGM 104
                               *  * :**   *:     .* .   :   .   . **: :    .:    :
                             MMMMMMMMMMMMMMMMMM4
Escherichia coli (EcoliMGST) SATWCALLLMVLANLWYMPWELVFSLR-- 131
Methylobacterium sp.         SIIWGVSVVGILLVLWGL----FVKILPY 136
Vibrio cholerae              QLTFGCLAVLMVLNLWVLPYSKFFYPLPT 133
                             .  :    : ::  ** :    ..

Mycobacterium sp.            ---------MTFALWMVLAAILLPYLTVMPAKVT---SRVDNHAPRRDMERLTGWQ-QRL 47
Acinetobacter baumannii      ------MHGISSIIYIILVACLLPYAFTVIAKFTGGFQPVDNQNPRDFLANTKGIA-ARA 53
Actinomyces slackii          MLPTILICVGTIAVSVVLGAHLVDLLFKVIDRTAK---PSSSGN-SVLLANEEKLGMHHG 56
                                       :  : ::* * *:     :  : :      ..      : .       :
Mycobacterium sp.            DWAHRNHFETFPAFAAAVIIAQMRGAPQDWADILAGLFVLIRLAYTGAYAADR------- 100
Acinetobacter baumannii      NAVQQNSFESLPLFLTAILMAEYMVVPEYFILRLGWAYIILRVIYGICYLSNW------- 106
Actinomyces slackii          MWIG-G-LERLAV--T--------------GLLLSGRYELLMGVIAIKVLAHWTGAKDSP 98
                                  . :* :    :                 *.  : ::         :.
Mycobacterium sp.            -----------PSLRSNLWSVGFLCILGLFALAAIGGPAAAPAG 133
Acinetobacter baumannii      -----------ATLRSIVWFLSLLCPIFLLIVTIKLT------- 135
Actinomyces slackii          ERPGAVERLTIGTLASLIWAVA--CGLAGKALLAWV-------- 132
                                         :* * :* :.  * :    :

Halorubrum trapanicum        MLLQTVTPVSVLGTTVLLALFLSIT---AHVAARNVLGDVDPRRALYIGPLPAVISVVGN 57
Nitrosopumilus sp.           MQS-F---NLFVGSACLLIVILALNVSRLRYKERVAHGYGDSKK------LRNAIVAHSN 50
Candidatus nitrosotenuis     ML--A---YICIGVLGFLVVGLGWGVSMLRLRRRIGIGAPDDPE----DPFFKLVRAHAN 51
                             *          :*   :* : *.      :   *   *  *  .      :   : . .*
Halorubrum trapanicum        ALELPAGLILLAALLV--DGTMFW-----WSYEQPRRLVAA------------------- 91
Nitrosopumilus sp.           ALEHLIPFALILYVLIAQNTSQTLVQGLAISFLVLRVIHALGVTRFVFRAWQVSSGLS-- 108
Candidatus nitrosotenuis     AAEY-APMLAVLMLVAGTPEPPAWVAMSMVCATAARLLHALGMVRSPSLARPHPLRFVGA 110
                             * *    :  :  ::               .    * : *
Halorubrum trapanicum        MTLIHAVVTTLLIGVLLLVSILLASMPG----- 119
Nitrosopumilus sp.           -------YALTIFSSCLILYNWL---------- 124
Candidatus nitrosotenuis     -------AGTYLFGALLALAVLLRGATGLGRPT 136
                                        ::.  * :   *
```

```
Anabaena cylindrica           ----MSPWTSLVTVLALIVYFVVTINVGRARFKYKISPPQTVGNPDFERVLRVQQNTLEQ  56
Synechocystis sp. PCC 6803    MTKTELLWPALITALATMLYLVLVINVGRARAKYGVMPPATTGNEDFERVLRVQYNTLEQ  60
Prochlorococcus marinus       --MTIPVLPAVVTLAAAIVYQGTMFAVAFARSQYKVKAPATSGPEEFERVLRVQQNTLEQ  58
                                       :::*  * ::*    : *. ** :* :  * * *  :******** *****

Anabaena cylindrica           LILFLPALWLFSIYISPIWASALGTAWVIGRITYAWGYYQAAEKRGIGFAISSLSSMGLI  116
Synechocystis sp. PCC 6803    LAFFLPGLWLFAIYRDPTIAAILGAVWLLGRILYAWGYYQAAEKRMVGFALGSLSSMILV  120
Prochlorococcus marinus       MMFFLPVFWLAALSSNTSVACLIGFIWVGARIAYGIGYWKAASLRGPGFAISLLASAVLL  118
                              : :*** :** ::  .   *. :*  *: .** *. **::**. *  ***:. *:*  *:

Anabaena cylindrica           LGSLIGIILDLVKLPGI 133
Synechocystis sp. PCC 6803    VGALLSILWQLRQLSQF 137
Prochlorococcus marinus       VMAIVGLFN-------- 127
                              : :::.::


Botrytis elliptica            MVTITLDADYGYVILAATSTFVLNFWHGINTGSYRKAAKIDYPAAYAPSSRT-------D  53
Alternaria atra               MAIIQIPSEYGYVLAACVSTFFVGIWHGGRVGGFRKAAKIPYPYEYASYEQVQTASPPSR  60
Massarina eburnea             MVSIELPAEYGYVLAATVSTFFVGTWLGMRVGMYRKQAKIPYPYEYASYEQIQTAEPSHA  60
                              *. * : ::****: * .***.:. * * ..* :** *** **  **  .:

Botrytis elliptica            TAAHQFNCAQRAHANFTENHSVAVAAMLVAGLEFPRSAAVLGGAWTISRWVYMRGYS-QG  112
Alternaria atra               AAMLAFNSAQRAHQNFNENHVTALGAMLITGLRYPVAAAVLGAVWSVNRVIYAIGYARTG  120
Massarina eburnea             KAMHEFNCAQRGHQNFNENHPIAIASFLITGLQYPRAAAILGTAWTVGRVLYALGYN-NG  119
                               *   **.***.* **.***  *:.::*::**.:* :**:** .*::.* :*  **   *

Botrytis elliptica            GVGGKGRYKGIWFWLFQMGLMGLCGFMGGKMVLEGKL  149
Alternaria atra               EDGGKGRYYGALGIIAHYVLVLMSGKAAYDLVMG---  154
Massarina eburnea             KEGGRGRYNGAFGMVAQYVSIIWAGVSAYGFIKAA--  154
                                **:*** *    : :   :  .*  .  ::


Arabidopsis thaliana          --------MAAITEFLPKEYGYVVLVLVFYCFLNLWMGAQVGRARKRYNVPYPTLYAIES  52
Citrus sinensis               MFSSIDSTVAAATQFLPTEYGYVALVLVFYCFLNFWMGFQVGAARKKYKVPYPTLYAVES  60
Brassica campestris           ---------MALTEFLPNEYGYVALVLVFYCFLNLWMGAQVGMARKRYNVPYPTLYAIES  51
                                        * *:***.*****.**********:*** *** ***:*:********:**

Arabidopsis thaliana          ENKDAKLFNCVQRGHQNSLEMMPMYFILMILGGMKHPCICTGLGLLYNVSRFFYFKGYAT  112
Citrus sinensis               ENKEAKLFNCVQRGHQNSLELMPVFFMLMIVGGLKHPCVCAVLGSLFTVTRFFYFKGYAT  120
Brassica campestris           ENKDAKLFNCVQRGHQNSLEMMPMYFVLMILGGMKHPCICAGLGLLYNITRFFYFKGYST  111
                              ***:****************:**::*:***:**:****:*: ** *:.::********:*

Arabidopsis thaliana          GDPMKRLTIGFVL----LSSYQCSLSLLIDLVMLVNRI 146
Citrus sinensis               GDPQKRLTIGKYGFLALLGLMVCTISFGINQLRG---- 154
Brassica campestris           GDPMKRLTIGKYGFLGLLGLMICTISFGVSLIRG---- 145
                              *** ******       *.   *::*: :. :
```

```
MGST2_DESRO     MAENSILLAAVSVLSVCQQGYFAVQVGKARLKYKVTPPAVYGSPEFERVFRAQQNCVEFY  60
MGST2_HUMAN     MAGNSILLAAVSILSACQQSYFALQVGKARLKYKVTPPAVTGSPEFERVFRAQQNCVEFY  60
MGST2_MONKEY    MAGNSILLAAVSILSACQQSYFALQVGKARLKYKVTPPAVTGSPEFERVFRAQQNCVEFY  60
                ** *********:**.***.***:**************** *******************

MGST2_DESRO     PIFLLTLWMAGWYFNQVFASCLGLVYIYARYQYFWGYSEAAKKRITGFRLSLGILALLTV 120
MGST2_HUMAN     PIFIITLWMAGWYFNQVFATCLGLVYIYGRHLYFWGYSEAAKKRITGFRLSLGILALLTL 120
MGST2_MONKEY    PIFIITLWMAGWYFNQVFATCLGLMYIYGRHLYFWGYSEAAKKRITGFRLSLGILALLTL 120
                ***::**************:****:***.*: ***************************:

MGST2_DESRO     LGTLGIADSFLDEYLDLNIAQKLRHF- 146
MGST2_HUMAN     LGALGIANSFLDEYLDLNIAKKLRRQF 147
MGST2_MONKEY    LGALGIANSFLDEYLDLNIAKKLRRQF 147
                **:****:************:***:


MGST3 SCHMA     MSLSKYISPSLTSLPLCLPRYYGGVILVGVSAFGLNLYFARRVMQARKEHNVELPIMYHP  60
MGST3 HUMAN     --------------MAVLSKEYGFVLLTGAASFIMVAHLAINVSKARKKYKVEYPIMYST  46
MGST3 MOUSE     --------------MAVLSKEYGFVLLTGAASFVMVLHLAINVGKARKKYKVEYPVMYST  46
                                 * : ** *:*.*.::* :  ::* .* :***:::** *:**

MGST3 SCHMA     T---DKLFNCIQRGHQNYLEVLPFFLMALFVGGLRYPRMYTACGAVFLLGRLLYFQGYAT 117
MGST3 HUMAN     DPENGHIFNCIQRAHQNTLEVYPPFLFFLAVGGVYHPRIASGLGLAWIVGRVLYAYGYYT 106
MGST3 MOUSE     DPENGHMFNCIQRAHQNTLEVYPPFLFFLTVGGVYHPRIASGLGLAWIIGRVLYAYGYYT 106
                    .::******.*** *** * **: * ***: :**: :. * .:::**:**  ** *

MGST3 SCHMA     GDPAKRYKGAVSMAGGFPMIMGLLVFGVQHLMASIRCTGCALRS--- 161
MGST3 HUMAN     GEPSKRSRGALGSIALLGLVGTTVCSAFQHLGWVKSGLGSGPKCCH- 152
MGST3 MOUSE     GDPSKRYRGAVGSLALFALMGTTVCSAFQHLGWIRPGLGYGSRSCHH 153
                *:*:** :**:.  . : ::   :  ..***       * . :.


MGST1 Rat       MADLKQLMDNEVLMAFTSYATIILAKMMFLSSATAFQRLTNKVFANPEDCAGFGKGENAK  60
MGST1 MARMOSET  MVDLSQLTDDEVFRAFASYATIILSKMMLMSAATAFYRMTRKVFANPEDCATFGKGENAK  60
MGST1 HUMAN     MVDLTQVMDDEVFMAFASYATIILSKMMLMSTATAFYRLTRKVFANPEDCVAFGKGENAK  60
                *.**.*: *:**: **:*******:***::*:**** *:*.*********. ********

MGST1 Rat       KFLRTDEKVERVRRAHLNDLENIVPFLGIGLLYSLSGPDLSTALIHFRIFVGARIYHTIA 120
MGST1 MARMOSET  KFLRTDDRVERVRRAHLNDLENIVPFLGIGLLYSLSGPDLSTAVLHFRIFVGARIYHTIA 120
MGST1 HUMAN     KYLRTDDRVERVRRAHLNDLENIIPFLGIGLLYSLSGPDPSTAILHFRLFVGARIYHTIA 120
                *:****::***************:*************** ***::***:***********

MGST1 Rat       YLTPLPQPNRGLAFFVGYGVTLSMAYRLLRSRLYL 155
MGST1 MARMOSET  YLTPLPQPNRALGFFVGYGVTLSMAYRLLKSRLYL 155
MGST1 HUMAN     YLTPLPQPNRALSFFVGYGVTLSMAYRLLKSKLYL 155
                **********.*.****************:*:***
```

```
mPGES-1 HUCHO        -------MLENEAFSCFVFYSVLLVIKMYVIAIITGQVRLRKKAFANPEDAQRHGGVQYY 53
mPGES-1 HUMAN        MPAHS-LVMSSPALPAFLLCSTLLVIKMYVVAIITGQVRLRKKAFANPEDALRHGGPQYC 59
mPGES-1 HORSE        MPPPSLAMVSGQALPAFLLCSTLLVIKMYAVAVITGQVRLRKKAFANPEDALRHGGLQFH 60
                            ::.. *: .*:: *.*******.:*:****************** **** *:

mPGES-1 HUCHO        RDDPDVERCRRAHRNDMENIFPFLFLGTVYSLIGPSLAVARAHFLVFFLFRVLHTVAYLC 113
mPGES-1 HUMAN        RSDPDVERCLRAHRNDMETIYPFLFLGFVYSFLGPNPFVAWMHFLVFLVGRVAHTVAYLG 119
mPGES-1 HORSE        RDDQDVERCLRAHRNDMETIYPFLFLGLVYSFLGPDPFVAQMHFLVFFLGRMVHTVAYLG 120
                     *.* ****** *********.*:****** ***::**.  **  *****:: *: ******

mPGES-1 HUCHO        VLRAPTRSLAYVVAQVPCVSMAVQVLAAVASYA 146
mPGES-1 HUMAN        KLRAPIRSVTYTLAQLPCASMALQILWEAARHL 152
mPGES-1 HORSE        KLRAPTRSLAYTVAQLPCASMALQIVWEAARHL 153
                      **** **::*.:**:**.***:*::  .* :

LTC4S ANGUILLA       MLDQVINIAAVTVLGVLEQAYFSLQVIYARRKYSVSPPSTTGPPEFERVFRAQVNCSEYF 60
LTC4S BOVIN          MKDEVALLASVTLLGVLLQAYFSLQVISARRAFRVSPPLTTGPPEFERIYRAQVNCSEYF 60
LTC4S HUMAN          MKDEVALLAAVTLLGVLLQAYFSLQVISARRAFRVSPPLTTGPPEFERVYRAQVNCSEYF 60
                     * *:* :*:**:**** ********* *** : **** *********::***********

LTC4S ANGUILLA       PIFICTLWVTGVFFSQAAASVCGLLYLYGRLMYFRGYSESAHSRLAPLYFSAKVLWLLIG 120
LTC4S BOVIN          PLFLAMLWVAGIFFHEGAAALCGLVYLFARLRYFQGYARSAQQRLAPLYASARALWLLVA 120
LTC4S HUMAN          PLFLATLWVAGIFFHEGAAALCGLVYLFARLRYFQGYARSAQLRLAPLYASARALWLLVA 120
                     *:*:. ***:*:** :.**::***:**:.** **:**:.**: ****** **:.****:.

LTC4S ANGUILLA       LSCLGVLNSMCQLYLGLDVLQQLSSFLLQN 150
LTC4S BOVIN          LAALGLLAHFLPAELRAALLGQLRKLLLRS 150
LTC4S HUMAN          LAALGLLAHFLPAALRAALLGRLRTLLPWA 150
                     *:.**:* :   *   :*  :*  .:*

FLAP AQUILA          MDQETLGSIVLLAIVTLISVVQNAFFASKLEHESKHCNGKGFQRAGSSAFDRVYTANQNC 60
FLAP HUMAN           MDQETVGNVVLLAIVTLISVVQNGFFAHKVEHESRTQNGRSFQRTGTLAFERVYTANQNC 60
FLAP RAT             MDQEAVGNVVLLAIVTLISVVQNAFFAHKVELESKAQSGRSFQRTGTLAFERVYTANQNC 60
                     ****::*.:*************.*** *:* **:  .*:.***:*: **:*********

FLAP AQUILA          GHAYPTFLAVLWCAGLLCSQAPAAFAGLMYLFVRQKYFVGYLGERTQSTPGYLFGKRIIL 120
FLAP HUMAN           VDAYPTFLAVLWSAGLLCSQVPAAFAGLMYLFVRQKYFVGYLGERTQSTPGYIFGKRIIL 120
FLAP RAT             VDAYPTFLVVLWTAGLLCSQVPAAFAGLMYLFVRQKYFVGYLGERTQSTPGYIFGKRIIL 120
                      .******.*** *******.*******************************:*******

FLAP AQUILA          FLFLMSVAGILNYYLIFFFGSDFEMYIKTITSAISPLLLIP 161
FLAP HUMAN           FLFLMSVAGIFNYYLIFFFGSDFENYIKTISTTISPLLLIP 161
FLAP RAT             FLFLMSLAGILNHYLIFFFGSDFENYIRTITTTISPLLLIP 161
                     ******:***:*:*********** **:**:::********
```

Figure S1. Multiple sequence alignment of representative prokaryotic and eukaryotic MAPEG proteins. In EcoliMGST, TM helices are boxed and refer to the *E. coli* sequence. Accession numbers are indicated in Figure 3. The conserved residues of the motif RX3NX2E/D are shown in red and bold. Proline residues are marked in red boxes. Asterisk (*), colon (:) and period (.) indicate identical, conserved substitution and semi-conserved substitution, respectively.
